# Plugging the Gaps: Catheter-Based Interventions in Elderly Patients Presenting With Pulmonary Embolism

**DOI:** 10.1016/j.jacadv.2025.101977

**Published:** 2025-07-23

**Authors:** Andrew Sharp, Hyeok-Hee Lee, Eric Secemsky

**Affiliations:** a School of Medicine, University College Dublin, Dublin, Ireland; b Department of Cardiology, Mater Misericordiae University Hospital, Dublin, Ireland; c Division of Cardiology, Harvard Medical School, Beth Israel Deaconess Medical Center, Boston, Massachusetts, USA; d Richard A. and Susan F. Smith Center for Outcomes Research in Cardiology, Beth Israel Deaconess Medical Center, Boston, Massachusetts, USA

**Keywords:** catheter interventions, elderly, pulmonary embolism, thrombectomy, thrombolysis

**Figure:**
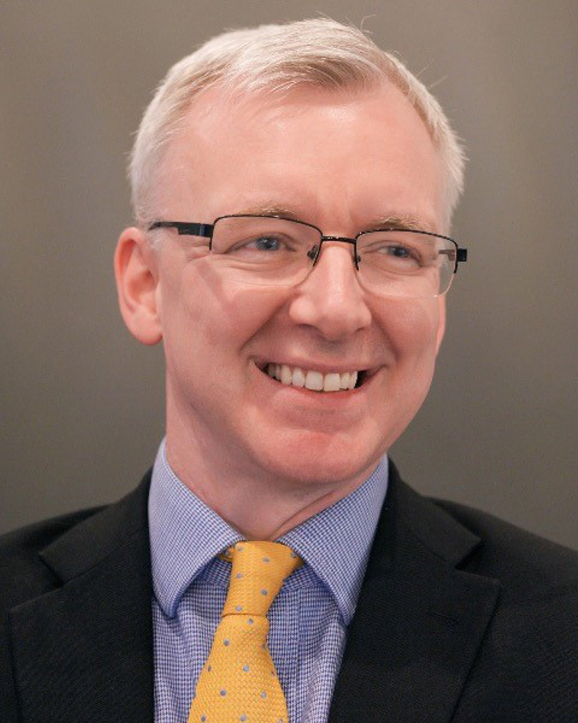

There are things in pulmonary embolism (PE) care that have widespread agreement. Firstly, patients presenting with PE should be anticoagulated as soon as possible to prevent secondary, and potentially life-threatening, sequelae of PE. Secondly, there are some patients with a higher risk of earlier decompensation and/or death than others. The European Society of Cardiology wrote what is widely accepted as being the definitive guidelines in this space in 2019,^1^ summarizing how to diagnose, risk stratify, and treat patients with PE, but much has changed in PE care in the last 6 years since this document was published, while much still remains unknown.

Intermediate-risk PE describes a patient with preserved blood pressure (BP) but one or more markers of right ventricular strain. The more ominous high-risk PE—that associated with obstructive shock—is defined as either that causing cardiac arrest or that causing compromise of end-organ function in association with evidence of BP compromise. Mortality rates for this condition range from 15% for those who have avoided cardiac arrest^2^ up to 65% in a Portuguese national survey of national outcomes from high-risk PE,^3^ incorporating those who have sustained cardiac arrest. The recommended treatment for high-risk PE is immediate reperfusion, and that has traditionally only been associated with 2 therapies, each of which has substantial limitations.

The first is systemic thrombolysis, either at “full dose,” typically 100 mg of alteplase over 2 hours for an adult of median body weight, or at half dose, with the aim of minimizing bleeding complications. The data that doing so avoids bleeding while maintaining efficacy are limited, and the basic principle is open to question—to bleed, one must have a potential bleeding source, in which case why would 50 mg of alteplase be substantially less likely to cause bleeding than 100 mg? It might, though, and the ongoing PEITHO 3 randomized controlled trial (RCT) examining “half dose” lytic against anticoagulation in intermediate-risk PE will help to clarify this.^4^

The conundrum of bleeding vs reperfusion goes through the minds of front-line clinicians regularly, leading to a substantial withholding of what should be considered life-saving thrombolysis for high-risk PE. Why would this occur when, without reperfusion, the mortality for high-risk PE may be over 50%?. A psychologist might say that, when reperfusion therapy is withheld, the disease is responsible for the outcome, whereas a therapy delivered that causes death by intracranial hemmorhage is thought by some to have died at the hands of the decision-maker. This has left clinicians in a situation whereby more than half of all patients with high-risk PE are treated only with heparin ± vasopressor/inotropic support, leaving a major gap in treatment for the sickest patients with PE. Surgical thrombectomy, involving cardiopulmonary bypass, has had many decades to fill this gap, but only a small minority are treated with this strategy.^5^

Secondly, this gap has increasingly been filled with catheter-based therapies (CBTs). One example is catheter-based thrombolysis, with lower doses of lytic delivered directly into the pulmonary arteries in the hope of maintaining efficacy at decreased bleeding risk. The theory is that smaller thrombolytic doses are immediately taken up by the clot and may result in lower circulating concentrations of lytic, especially given protocols incorporating doses as low as 8 to 12 mg of alteplase.^6^ However, there remain 2 concerns with catheter lysis for high-risk PE. First, to leverage the theoretical lower risks of bleeding from catheter lysis, response time may be delayed and leave patients waiting in a potentially deadly hemodynamic spiral of shock. Secondly, the bleeding concerns remain, albeit at a magnitude smaller compared with systemic lytics.

Herewith enters catheter thrombectomy, a basic concept given new life by the advent of new technologies. The U.S. Food and Drug Administration has now approved 3 technologies designed to aspirate clot from the pulmonary circulation: the Inari Flowtriever, with sizes from 16- to 24-F^7^ the Penumbra Indigo system, with sizes from 12- to 16-F,^8^ and the Alphavac system, currently available in 18-F.^9^ The promise of these technologies is two-fold: early/immediate hemodynamic improvement on the table without the requirement for lytics and a theoretically lower bleeding risk. Venous access site/cardiopulmonary complications occasionally occur given the size of the catheters, but the possibility of immediate reperfusion with lower bleeding risk—certainly when compared to systemic lysis—offers a new option for unstable PE patients, and these devices now represent the majority of catheter-based interventions performed for acute PE.

Importantly, randomized clinical trials are underway to assess all catheter-based options in PE, and only a few have been completed to date. The first trial performed—the ULTIMA trial—randomized intermediate-risk patients to catheter lysis or anticoagulation, with a 24-hour powered endpoint showing improvement in right ventricle:left ventricle diameter ratio by echocardiography with catheter treatment.^10^ Since that time, we have yet to see a powered RCT of catheters vs anticoagulation. However, HI PEITHO will be among the first to report testing ultrasound-assisted, catheter-directed thrombolysis vs anticoagulation in intermediate-high-risk PE^11^ and will be followed by PEERLESS II, testing Flowtriever vs anticoagulation in an intermediate-risk population.^12^ The NIH sponsored PE TRACT will assess VO_2_ max on cardiopulmonary exercise testing at 3 months and NYHA functional class at 1 year following catheter-based treatment (either catheter-based lysis or thrombectomy) vs anticoagulation in intermediate-risk PE. In the high-risk PE space, the PERSEVERE and TORPEDO trials will randomize patients to catheter thrombectomy vs medical therapy (including systemic lysis) for those in shock.

So what is the current role for observational data when RCTs are so close to completion? The pyramid of evidence typically shows RCTs sitting above observational data, but can we close the gap in data quality, and, perhaps more importantly, can large-scale observational data fill in gaps that RCTs inevitably leave behind? One much-needed area with a data gap is age. Several of the landmarks described have upper age limits, and many trials exclude patients who have sustained cardiac arrest or whose comorbidities preclude enrollment with long-term horizons in mind. The only option we have in this space is observational data with novel statistical resources used to narrow the evidence gap.

The manuscript by Watanabe^13^ analyzes patients within the U.S. Medicare system who received catheter-based treatment for PE, and all described are between the ages of 65 and 99 years. We already see originality in the dataset, as few 90-year-olds are likely to be enrolled in landmark RCTs, yet the life expectancy of an average 90-year-old without PE is approximately 4 to 5 years by Social Security Life Table analysis and should remain a candidate for treatment where appropriate. As with all observational data, though, we have a question of unmeasured confounders—if patients do better following catheter treatment, is that because of catheter treatment or because they were deemed robust enough to be considered for treatment?

The current manuscript proposes a mortality impact for patients receiving catheter-based treatment and attempts to deal with unmeasured confounders by 2 major statistical mechanisms. The first is through propensity score matching weights, whereby patient cohorts are matched not only purely by finding patients who did/did not receive the intervention in question but also by weighting those who could have received the therapy but did not. Thus, this attempts to account for treatment selection bias. The second is an instrumental variable analysis, whereby natural randomization can be leveraged based on purely situation circumstances. An example is for patients who live in different geographies. If patients live closer to an experienced CBT hospital, they may be more likely to receive CBT than those who live further away. By looking at treatment differences generated by geography alone, one can account for both measurable and nonmeasurable biases among those receiving the therapy and those not. The aim is to mimic an RCT in patients who are difficult to ever include in RCTs and thus have a new class of data analysis that is not easy to replicate through the traditional pyramid of evidence (Figure 1).

**Figure 1:**
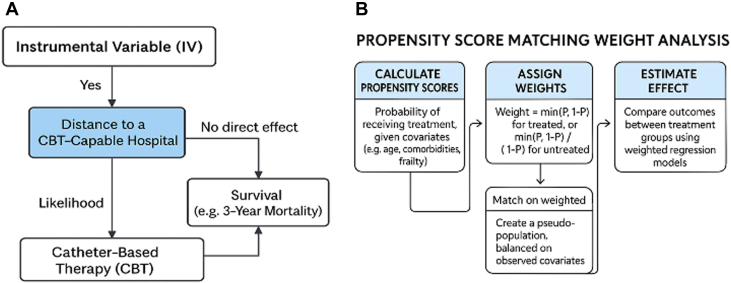
Statistical Tools for Minimizing Confounding Within Observational Dataset Analysis Understanding (A) IV analysis and (B) propensity score matching weight analysis: tools for minimizing unmeasured confounders in observational datasets. CBT = catheter-based therapy; IV = instrumental variable.

The findings of improved short- and long-term mortality in those receiving CBT may still be confounded, but the methods used are about as good as it currently gets for inferring benefit in potentially excluded RCT groups. The finding of higher bleeding complications with CBT also passes “the sniff test” for congruent data—bleeding complications tracked the more invasive therapy strategies.

In intermediate-risk PE, the authors describe uncertain short-term mortality findings but better long-term outcomes. The early signal could still be suppressed through unmeasured confounding despite the methods employed. Alternatively, it may be that modern medicine is increasingly good at preserving patients with preserved BP through all-round better care, and so the low early event rate left little room for improvement. We know that up to half of patients at 1 year describe persistent physical/mental sequelae of their index PE, and whatever the mechanism of the “post-PE syndrome,” earlier offloading of the right ventricle ± earlier, healthier mobilization of the patient could produce a long-term signal through several physiological mechanisms outside of mortality alone.^14^

The data propose a signal of an intuitive and plausible concept—that offloading a right ventricle under strain and improving pulmonary perfusion at an earlier point in the disease process could provide short- and long-term improvements in health, especially when risks are high. Other cardiovascular technologies have, however, shown signals of benefit in observational data that have not subsequently been borne out in RCTs. The role for this manuscript, therefore, seems to be: if benefit is proven in younger, healthier patients in RCTs, what of older patients? Perhaps the current manuscript describes the data that could plug that gap.

## Funding support and author disclosures

Dr Sharp is a consultant and receives speaker fees from Medtronic, Philips, Boston Scientific, Penumbra, Angiodynamics, and Recor Medical and has stock options in Althea Medical. Dr Secemsky has received funding from 10.13039/100000050NIH/NHLBIK23HL150290; grants to the institution from BD, Boston Scientific, Cook, Medtronic, and Philips; and is a consultant for Abbott, Asahi, BD, Boston Scientific, Conavi, Cook, Cordis, Endovascular Engineering, Evident Vascular, Gore, InfraRedx, Medtronic, Penumbra, Philips, RapidAI, RampartIC, R3, Regeneron, Shockwave, Siemens, SoniVie, Teleflex, Terumo, Thrombolex, VentureMed, and Zoll. Dr Lee has reported that he has no relationships relevant to the contents of this paper to disclose.
